# Crystal structure and Hirshfeld surface analysis of (*E*)-1-[(4,7-di­methyl­quinolin-2-yl)methyl­idene]semicarbazide dihydrate

**DOI:** 10.1107/S2056989018014925

**Published:** 2018-10-31

**Authors:** Ercan Aydemir, Sevgi Kansiz, Necmi Dege, Hasan Genc, Snizhana V. Gaidai

**Affiliations:** a Ondokuz Mayıs University, Faculty of Arts and Sciences, Department of Chemistry, 55139, Samsun, Turkey; b T.R. Ministry of Forestry and Water Affairs, 11th Regional Directorate, 55030, Ilkadım-Samsun, Turkey; c Ondokuz Mayıs University, Faculty of Arts and Sciences, Department of Physics, 55139, Kurupelit, Samsun, Turkey; d Van Yüzüncü Yıl University, Faculty of Education, Department of Sciences, Van, Turkey; e Taras Shevchenko National University of Kyiv, Department of Chemistry, 64, Vladimirska Str., Kiev 01601, Ukraine

**Keywords:** crystal structure, semicarbazone, Hirshfeld surface

## Abstract

In the title compound, C_13_H_14_N_4_O·2H_2_O, the organic mol­ecule is almost planar. In the crystal, the mol­ecules are linked by O—H⋯O, N—H⋯O and O—H⋯N hydrogen bonds, forming a two-dimensional network parallel to (10). Hirshfeld surface analysis and two-dimensional fingerprint plots indicate that the most important contributions to the crystal packing are from H⋯H (55.4%), H⋯O/O⋯H (14.8%), H⋯C/C⋯H (11.7%) and H⋯N/N⋯H (8.3%) inter­actions.

## Abstract 2

The semicarbazone derivative mol­ecule is almost planar. In the crystal, the mol­ecules are linked by O—H⋯O, N—H⋯O and O—H⋯N hydrogen bonds.

## Chemical context   

Semicarbazones are important inter­mediates in organic synthesis, mainly for obtaining heterocyclic rings such as oxa­diazo­les and pyrazolidones (Arfan & Rukiah, 2015 ▸). Furthermore, they are used for the isolation, purification and characterization of aldehydes and ketones as well as for the protection of carbonyl groups. They possess a wide range of bioactivities and pharmacological applications (Jadon *et al.*, 2011 ▸). The chemistry of semicarbazones is inter­esting because of their special role in biological applications, exhibiting anti-proliferative, anti-tumoral, anti­convulsant, anti-trypanosomal, herbicidal and biocidal activities (Arfan & Rukiah, 2015 ▸). Beside these, a number of semicarbazones have also been reported to possess anti­fungal, anti­bacterial and anti­tubercular activities (Jadon *et al.*, 2011 ▸). Semicarbazones are commonly used as ligands in coordination chemistry and are biologically active compounds. Their complexation with different metals increases the bioactivity of these mol­ecules (Nasrullah *et al.*, 2013 ▸, Afrasiabi *et al.*, 2005 ▸).

Semicarbazones exist predominantly in the amido form in the solid state whereas due to the inter­actions of the solvent mol­ecules they can exhibit a amido–iminol tautomerism in solution state (Casas *et al.*, 2000 ▸).
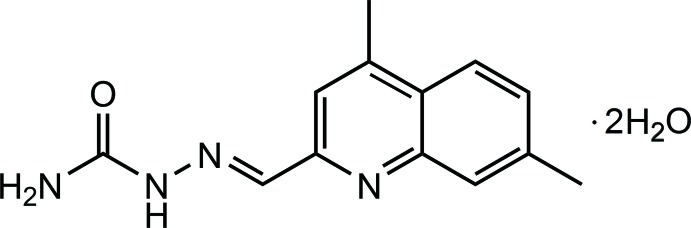


## Structural commentary   

**Figure 1:**
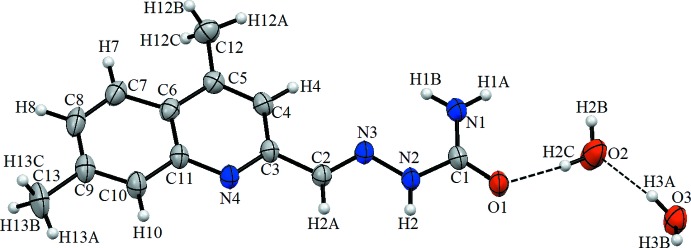
The mol­ecular structure of the title compound, showing the atom labelling. Displacement ellipsoids are drawn at the 20% probability level.

In the title compound (Fig. 1 ▸), the C3–C6/C11/N4 ring [r.m.s. deviation 0.0054 Å, maximum deviation of 0.0080 (12) Å for N4] is inclined to the C6–C11 aromatic ring by 1.75 (8)°. While these rings are almost co-planar, the N2—N3—C2—C3 torsion angle of −179.41 (16)° also indicates the general planarity of the mol­ecule. The aromatic C—C distances for the title compound range from 1.356 (3) Å to 1.500 (3) Å. The C2–N3 bond length [1.272 (2) Å] is in agreement with that for a double bond. The C1—N1 [1.316 (2) Å] and C3—N4 [1.319 (2) Å] bond lengths are essentially the same, as are the C1—N2 and C11—N4 distances[1.360 (2) and 1.372 (2) Å, respectively]. The organic mol­ecule and the two water mol­ecules in the asymmetric unit are linked by O—H⋯O hydrogen bonds (Fig. 1 ▸ and Table 1 ▸).

## Supra­molecular features   

**Figure 2:**
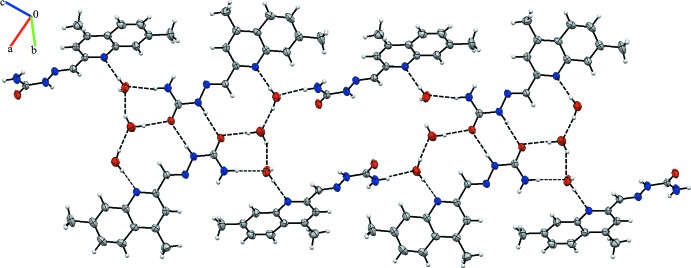
A view of the crystal packing of the title compound. Dashed lines denote hydrogen bonds (Table 1 ▸).

The crystal packing of the title compound features four inter­molecular (O—H⋯O, N—H⋯O and O—H⋯N) hydrogen bonds (Table 1 ▸ and Fig. 2 ▸) as well as those already mentioned, forming a two-dimensional network parallel to (10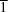). All three O atoms of the compound are involved in hydrogen bonds.

## Hirshfeld surface analysis   

**Figure 3:**
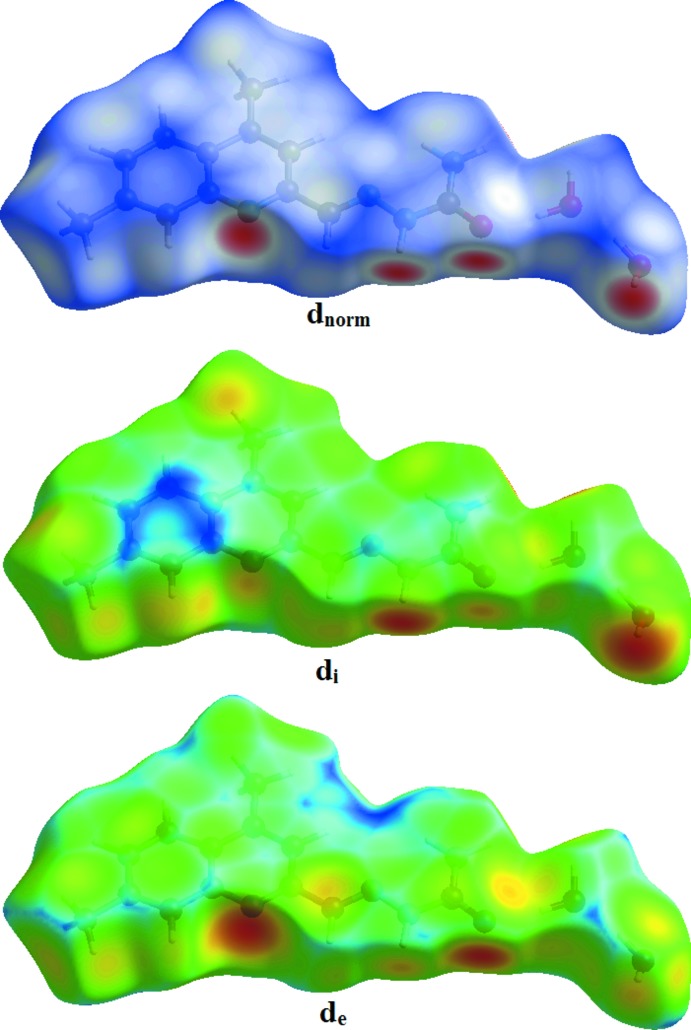
The Hirshfeld surfaces of the title compound mapped over *d*_norm_, *d*_i_ and *d*_e_.

Hirshfeld surface was used to investigate and qu­antify the inter­molecular inter­actions in the title structure (*CrystalExplorer*; Turner *et al.*, 2017 ▸). The Hirshfeld surfaces were plotted using a standard (high) surface resolution with the three-dimensional *d_norm_* surfaces mapped over a fixed colour scale of −0.578 (red) to 1.362 (blue) a.u. The red spots on the surfaces indicate the inter­molecular contacts involved in the hydrogen bonds (Sen *et al.*, 2018 ▸; Kansiz *et al.*, 2018 ▸; Gümüş *et al.*, 2018 ▸). Those in Figs. 3 ▸ and 4 ▸ correspond to the near-type H⋯O and H⋯N contacts resulting from O—H⋯O, N—H⋯O and O—H⋯N hydrogen bonds (Table 1 ▸).

**Figure 5:**
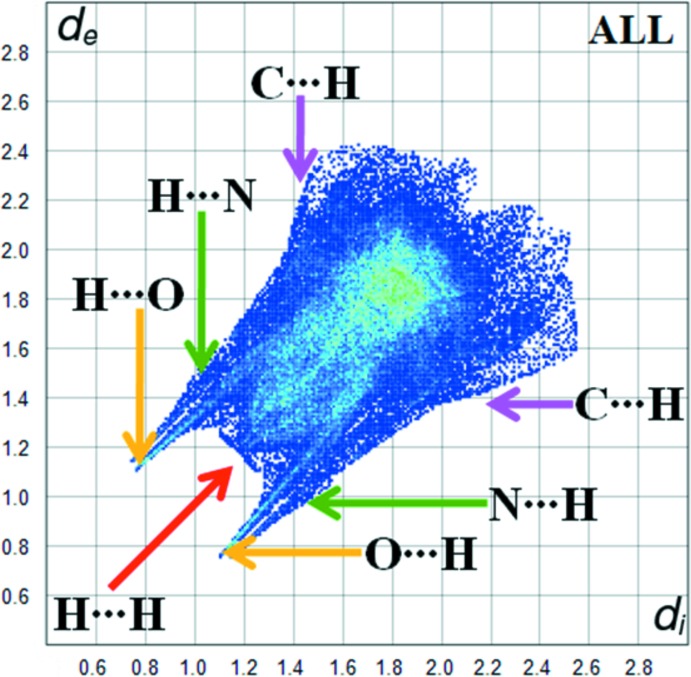
The overall fingerprint plot for the title compound.

**Figure 6:**
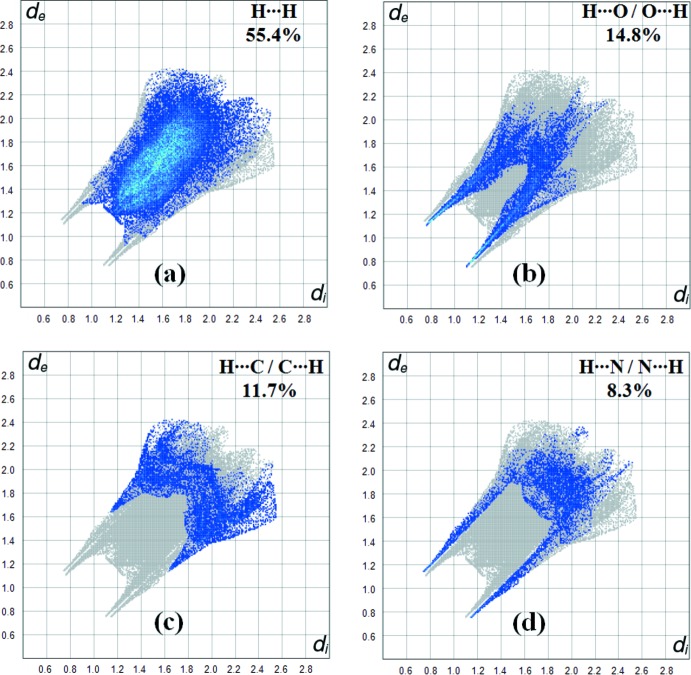
Two-dimensional fingerprint plots with a *d*_norm_ view of the (*a*) H⋯H (55.4%), (*b*) H⋯O/O⋯H (14.8%), (*c*) H⋯C/C⋯H (11.7%) and (*d*) H⋯N/N⋯H (8.3%) contacts in the title compound.

Fig. 5 ▸ shows the two-dimensional fingerprint of the sum of the contacts contributing to the Hirshfeld surface represented in normal mode. Fig. 6 ▸ *a* (H⋯H) shows the two-dimensional fingerprint of the (*d*_i_, *d*_e_) points associated with hydrogen atoms. It is characterized by an end point that points to the origin and corresponds to *d*_i_ = *d*_e_ = 1.2 Å, which indicates the presence of the H⋯H contacts in this study (55.4%). Fig. 6 ▸ *b* represents the O⋯H/H⋯O contacts (14.8%) between the oxygen atoms inside the surface and the hydrogen atoms outside the surface and has two symmetrical points at the top, bottom left and right, *d*_e_ + *d*_i_ = 1.9 Å. These data are characteristic of O—H⋯O and N—H⋯O hydrogen bonds. Fig. 6 ▸ *c* shows the contacts (C⋯H/H⋯C = 11.7%) between the carbon atoms inside the surface and the hydrogen atoms outside the surface of Hirshfeld and *vice versa*. There are two symmetrical wings on the left and right sides. In Fig. 6 ▸ *d*, the two symmetrical points at the top, bottom left and right, *d*_e_ + *d*_i_ = 1.8 Å, indicate the presence of H⋯N/N⋯H (8.3%) contacts. These data are characteristic of O—H⋯N hydrogen bonds (Table 1 ▸).

## Synthesis and crystallization   

The title compound was synthesised following a reported procedure by (Aydemir & Kaban, 2018 ▸). A hot ethano­lic solution (5 mL) of of semicarbazide hydro­chloride (1 mmol) and (0.1 mol) of sodium acetate trihidrate (1.5 mmol) in 2 mL water was slowly added to a solution of 2,7-di­methyl­quinoline-2-carboxaldehyde (1.0 mmol) in 10 mL of hot ethanol. The mixture was refluxed on a steam bath for 2 h until the colour changed. On completion of the reaction (monitored by TLC) the mixture was allowed to cool to room temperature. The separated solid was filtered and washed with cold water, ethanol and diethyl ether and then single crystals suitable for X-ray diffraction analysis were grown by slow evaporation of a saturated solution of the resultant compound in acetonitrile; colourless prismatic crystals were obtained in 83% yield, m.p. 503.5 K (deca­ying).

## Refinement   

Crystal data, data collection and structure refinement details are summarized in Table 2 ▸. C-bound H atoms were geometrically positioned with C—H distances of 0.93–0.96 Å. and refined as riding, with *U*_iso_(H) = 1.2*U*_eq_(C). N-bound H atoms were located in difference-Fourier maps and refined isotropically. The water H atoms were located in a difference-Fourier map and refined isotropically subject to a restraint of O—H = 0.85±2 Å.

## Supplementary Material

Crystal structure: contains datablock(s) I. DOI: 10.1107/S2056989018014925/xu5942sup1.cif

Structure factors: contains datablock(s) I. DOI: 10.1107/S2056989018014925/xu5942Isup2.hkl

Supporting information file. DOI: 10.1107/S2056989018014925/xu5942Isup3.cml

CCDC reference: 1865663

Additional supporting information:  crystallographic information; 3D view; checkCIF report

## Figures and Tables

**Figure 4:**
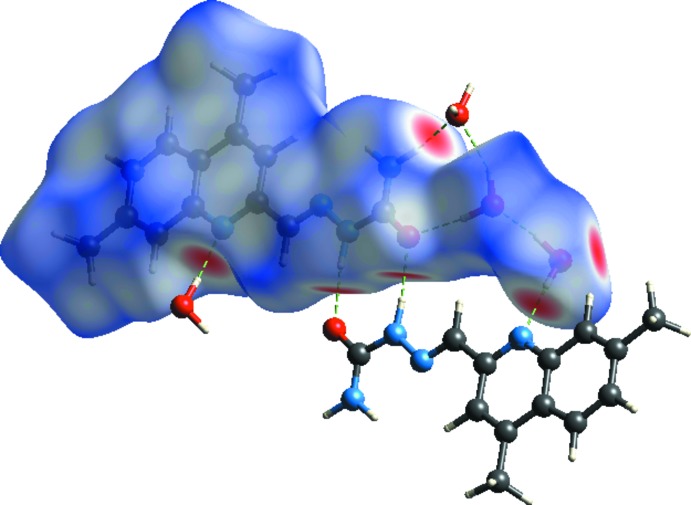
Hirshfeld surfaces mapped over *d*_norm_ to visualize the inter­molecular inter­actions.

**Table 1:** Hydrogen-bond geometry (Å, °)

*D*—H⋯*A*	*D*—H	H⋯*A*	*D*⋯*A*	*D*—H⋯*A*
N1—H1*A*⋯O3^i^	0.86	2.08	2.9277 (18)	171
N2—H2⋯O1^ii^	0.86	2.01	2.867 (2)	175
O2—H2*B*⋯O3^i^	0.85	2.03	2.814	154
O2—H2*C*⋯O1	0.85	1.92	2.769	175
O3—H3*A*⋯O2	0.85	1.83	2.665	169
O3—H3*B*⋯N4^ii^	0.85	2.02	2.8706 (1)	176

Symmetry codes: (i) 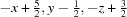; (ii) 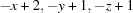.

**Table 2:** Experimental details

Crystal data	
Chemical formula	C_13_H_14_N_4_O·2H_2_O
*M*_r_	278.31
Crystal system, space group	Monoclinic, *P*2_1_/*n*
Temperature (K)	296
*a*, *b*, *c* (Å)	10.4731 (7), 7.4612 (5), 18.4906 (14)
β (°)	94.201 (6)
*V* (Å^3^)	1441.01 (18)
*Z*	4
Radiation type	Mo *K*α
μ (mm^−1^)	0.09
Crystal size (mm)	0.72 × 0.41 × 0.25

Data collection	
Diffractometer	Stoe IPDS 2
Absorption correction	Integration (*X-RED32*; Stoe & Cie, 2002 ▸)
*T*_min_, *T*_max_	0.953, 0.984
No. of measured, independent and observed [*I* > 2σ(*I*)] reflections	9088, 2981, 1564
*R*_int_	0.049
(sin θ/λ)_max_ (Å^−1^)	0.628

Refinement	
*R*[*F* ^2^ > 2σ(*F* ^2^)], *wR*(*F* ^2^), *S*	0.043, 0.099, 0.86
No. of reflections	2981
No. of parameters	189
H-atom treatment	H-atom parameters constrained
Δρ_max_, Δρ_min_ (e Å^−3^)	0.21, −0.17

Computer programs: *X-AREA* and *X-RED32* (Stoe & Cie, 2002 ▸), *SHELXT2017* (Sheldrick, 2015*a* ▸), *SHELXL2017* (Sheldrick, 2015*b* ▸), *ORTEP-3 for Windows* and *WinGX* (Farrugia, 2012 ▸) and *PLATON* (Spek, 2009 ▸).

## supplementary crystallographic information

### Crystal data

**Table:**

C_13_H_14_N_4_O·2H_2_O	*F*(000) = 592
*M**_r_* = 278.31	*D*_x_ = 1.283 Mg m^−^^3^
Monoclinic, *P*2_1_/*n*	Mo *K*α radiation, λ = 0.71073 Å
*a* = 10.4731 (7) Å	Cell parameters from 7023 reflections
*b* = 7.4612 (5) Å	θ = 2.0–29.9°
*c* = 18.4906 (14) Å	µ = 0.09 mm^−^^1^
β = 94.201 (6)°	*T* = 296 K
*V* = 1441.01 (18) Å^3^	Prism, colorless
*Z* = 4	0.72 × 0.41 × 0.25 mm

### Data collection

**Table:**

Stoe IPDS 2 diffractometer	2981 independent reflections
Radiation source: sealed X-ray tube, 12 x 0.4 mm long-fine focus	1564 reflections with *I* > 2σ(*I*)
Detector resolution: 6.67 pixels mm^-1^	*R*_int_ = 0.049
rotation method scans	θ_max_ = 26.5°, θ_min_ = 2.2°
Absorption correction: integration (X-RED32; Stoe & Cie, 2002)	*h* = −13→13
*T*_min_ = 0.953, *T*_max_ = 0.984	*k* = −9→7
9088 measured reflections	*l* = −23→23

### Refinement

**Table:**

Refinement on *F*^2^	0 restraints
Least-squares matrix: full	Hydrogen site location: mixed
*R*[*F*^2^ > 2σ(*F*^2^)] = 0.043	H-atom parameters constrained
*wR*(*F*^2^) = 0.099	*w* = 1/[σ^2^(*F*_o_^2^) + (0.0475*P*)^2^] where *P* = (*F*_o_^2^ + 2*F*_c_^2^)/3
*S* = 0.86	(Δ/σ)_max_ < 0.001
2981 reflections	Δρ_max_ = 0.21 e Å^−^^3^
189 parameters	Δρ_min_ = −0.16 e Å^−^^3^

### Special details

**Table:**

Geometry. All esds (except the esd in the dihedral angle between two l.s. planes) are estimated using the full covariance matrix. The cell esds are taken into account individually in the estimation of esds in distances, angles and torsion angles; correlations between esds in cell parameters are only used when they are defined by crystal symmetry. An approximate (isotropic) treatment of cell esds is used for estimating esds involving l.s. planes.

### Fractional atomic coordinates and isotropic or equivalent isotropic displacement parameters (Å^2^)

**Table:**

	*x*	*y*	*z*	*U*_iso_*/*U*_eq_	
O1	1.05531 (11)	0.4361 (2)	0.59020 (6)	0.0639 (4)	
N4	0.46860 (13)	0.2933 (2)	0.41455 (7)	0.0520 (4)	
O3	1.41681 (15)	0.7083 (2)	0.72180 (7)	0.0809 (5)	
H3A	1.358985	0.628748	0.723513	0.121*	
H3B	1.449265	0.703015	0.681041	0.121*	
N3	0.73918 (13)	0.3228 (2)	0.53869 (7)	0.0512 (4)	
N2	0.86130 (13)	0.3854 (2)	0.53533 (8)	0.0563 (4)	
H2	0.884164	0.432981	0.495938	0.068*	
N1	0.90821 (15)	0.2915 (2)	0.65190 (8)	0.0666 (5)	
H1A	0.959716	0.280022	0.690028	0.080*	
H1B	0.831568	0.249905	0.651516	0.080*	
C1	0.94666 (17)	0.3726 (3)	0.59415 (9)	0.0513 (5)	
C3	0.53144 (15)	0.2811 (2)	0.47886 (9)	0.0478 (5)	
C11	0.34215 (16)	0.2435 (3)	0.40929 (10)	0.0514 (5)	
O2	1.21793 (18)	0.4846 (3)	0.71357 (9)	0.1137 (7)	
H2B	1.196227	0.406526	0.743827	0.170*	
H2C	1.169860	0.475672	0.674650	0.170*	
C4	0.47587 (17)	0.2176 (3)	0.54060 (10)	0.0533 (5)	
H4	0.524750	0.211872	0.584605	0.064*	
C5	0.35095 (17)	0.1640 (2)	0.53701 (9)	0.0526 (5)	
C2	0.66482 (16)	0.3413 (3)	0.48165 (9)	0.0507 (5)	
H2A	0.695772	0.394234	0.440880	0.061*	
C6	0.27988 (16)	0.1782 (2)	0.46867 (10)	0.0515 (5)	
C10	0.27452 (18)	0.2618 (3)	0.34094 (10)	0.0636 (6)	
H10	0.317254	0.303577	0.301931	0.076*	
C7	0.14862 (18)	0.1346 (3)	0.45653 (12)	0.0661 (6)	
H7	0.104435	0.091112	0.494685	0.079*	
C9	0.14763 (19)	0.2196 (3)	0.33076 (12)	0.0662 (6)	
C12	0.2915 (2)	0.0934 (3)	0.60264 (11)	0.0734 (6)	
H12A	0.354999	0.088246	0.642736	0.110*	
H12B	0.258178	−0.024666	0.592665	0.110*	
H12C	0.223136	0.171292	0.614533	0.110*	
C8	0.08617 (19)	0.1551 (3)	0.39019 (14)	0.0737 (7)	
H8	−0.000175	0.125376	0.384002	0.088*	
C13	0.0751 (2)	0.2446 (4)	0.25749 (13)	0.0981 (9)	
H13A	0.128900	0.304520	0.225274	0.147*	
H13B	−0.000100	0.315338	0.263038	0.147*	
H13C	0.050553	0.129586	0.237730	0.147*	

### Atomic displacement parameters (Å^2^)

**Table:**

	*U*^11^	*U*^22^	*U*^33^	*U*^12^	*U*^13^	*U*^23^
O1	0.0413 (7)	0.0946 (11)	0.0541 (8)	−0.0070 (7)	−0.0073 (6)	0.0052 (7)
N4	0.0431 (8)	0.0643 (11)	0.0476 (8)	−0.0022 (7)	−0.0039 (7)	−0.0041 (7)
O3	0.0725 (10)	0.1144 (14)	0.0546 (8)	−0.0238 (9)	−0.0030 (7)	−0.0119 (8)
N3	0.0423 (8)	0.0593 (11)	0.0507 (8)	−0.0027 (7)	−0.0040 (7)	−0.0028 (7)
N2	0.0411 (8)	0.0792 (12)	0.0473 (8)	−0.0071 (8)	−0.0054 (7)	0.0062 (8)
N1	0.0537 (9)	0.0907 (14)	0.0534 (9)	−0.0120 (9)	−0.0100 (7)	0.0138 (9)
C1	0.0453 (11)	0.0588 (13)	0.0485 (10)	0.0021 (9)	−0.0067 (8)	−0.0030 (9)
C3	0.0446 (10)	0.0517 (12)	0.0462 (10)	−0.0008 (8)	−0.0036 (8)	−0.0055 (8)
C11	0.0424 (10)	0.0546 (13)	0.0560 (11)	−0.0008 (9)	−0.0044 (8)	−0.0088 (9)
O2	0.1052 (13)	0.164 (2)	0.0684 (10)	−0.0673 (13)	−0.0153 (10)	0.0070 (11)
C4	0.0527 (11)	0.0583 (13)	0.0481 (10)	0.0030 (9)	−0.0030 (8)	−0.0031 (9)
C5	0.0520 (11)	0.0513 (12)	0.0548 (10)	0.0021 (9)	0.0057 (9)	−0.0027 (9)
C2	0.0451 (10)	0.0591 (12)	0.0471 (9)	−0.0022 (9)	−0.0015 (8)	−0.0027 (8)
C6	0.0455 (10)	0.0461 (12)	0.0629 (11)	−0.0018 (9)	0.0041 (9)	−0.0066 (9)
C10	0.0510 (11)	0.0831 (16)	0.0553 (11)	−0.0025 (10)	−0.0063 (9)	−0.0080 (10)
C7	0.0488 (11)	0.0660 (15)	0.0835 (14)	−0.0096 (10)	0.0060 (10)	−0.0029 (11)
C9	0.0500 (11)	0.0723 (15)	0.0740 (14)	−0.0004 (11)	−0.0113 (11)	−0.0139 (12)
C12	0.0686 (13)	0.0830 (17)	0.0703 (13)	−0.0015 (12)	0.0163 (11)	0.0085 (11)
C8	0.0430 (11)	0.0739 (16)	0.1022 (17)	−0.0071 (10)	−0.0098 (12)	−0.0148 (13)
C13	0.0682 (15)	0.128 (2)	0.0921 (17)	0.0009 (14)	−0.0359 (13)	−0.0158 (15)

### Geometric parameters (Å, º)

**Table:**

O1—C1	1.240 (2)	C4—H4	0.9300
N4—C3	1.3193 (19)	C5—C6	1.423 (2)
N4—C11	1.372 (2)	C5—C12	1.500 (3)
O3—H3A	0.8500	C2—H2A	0.9300
O3—H3B	0.8500	C6—C7	1.414 (3)
N3—C2	1.2719 (19)	C10—C9	1.365 (3)
N3—N2	1.3673 (19)	C10—H10	0.9300
N2—C1	1.360 (2)	C7—C8	1.356 (3)
N2—H2	0.8600	C7—H7	0.9300
N1—C1	1.316 (2)	C9—C8	1.399 (3)
N1—H1A	0.8600	C9—C13	1.515 (3)
N1—H1B	0.8600	C12—H12A	0.9600
C3—C4	1.401 (2)	C12—H12B	0.9600
C3—C2	1.465 (2)	C12—H12C	0.9600
C11—C6	1.405 (3)	C8—H8	0.9300
C11—C10	1.409 (2)	C13—H13A	0.9600
O2—H2B	0.8500	C13—H13B	0.9600
O2—H2C	0.8501	C13—H13C	0.9600
C4—C5	1.365 (2)		
			
C3—N4—C11	117.43 (16)	C11—C6—C7	117.21 (17)
H3A—O3—H3B	109.5	C11—C6—C5	118.53 (15)
C2—N3—N2	116.30 (15)	C7—C6—C5	124.24 (19)
C1—N2—N3	120.14 (16)	C9—C10—C11	121.4 (2)
C1—N2—H2	119.9	C9—C10—H10	119.3
N3—N2—H2	119.9	C11—C10—H10	119.3
C1—N1—H1A	120.0	C8—C7—C6	121.1 (2)
C1—N1—H1B	120.0	C8—C7—H7	119.5
H1A—N1—H1B	120.0	C6—C7—H7	119.5
O1—C1—N1	124.08 (15)	C10—C9—C8	118.05 (18)
O1—C1—N2	118.59 (17)	C10—C9—C13	120.9 (2)
N1—C1—N2	117.33 (17)	C8—C9—C13	121.01 (19)
N4—C3—C4	123.22 (15)	C5—C12—H12A	109.5
N4—C3—C2	114.97 (16)	C5—C12—H12B	109.5
C4—C3—C2	121.80 (14)	H12A—C12—H12B	109.5
N4—C11—C6	122.64 (15)	C5—C12—H12C	109.5
N4—C11—C10	117.17 (18)	H12A—C12—H12C	109.5
C6—C11—C10	120.19 (16)	H12B—C12—H12C	109.5
H2B—O2—H2C	109.5	C7—C8—C9	122.02 (18)
C5—C4—C3	120.88 (15)	C7—C8—H8	119.0
C5—C4—H4	119.6	C9—C8—H8	119.0
C3—C4—H4	119.6	C9—C13—H13A	109.5
C4—C5—C6	117.28 (17)	C9—C13—H13B	109.5
C4—C5—C12	121.14 (16)	H13A—C13—H13B	109.5
C6—C5—C12	121.58 (17)	C9—C13—H13C	109.5
N3—C2—C3	121.35 (17)	H13A—C13—H13C	109.5
N3—C2—H2A	119.3	H13B—C13—H13C	109.5
C3—C2—H2A	119.3		
			
C2—N3—N2—C1	179.33 (17)	N4—C11—C6—C5	0.3 (3)
N3—N2—C1—O1	−177.53 (16)	C10—C11—C6—C5	−179.15 (17)
N3—N2—C1—N1	2.9 (3)	C4—C5—C6—C11	0.9 (3)
C11—N4—C3—C4	1.1 (3)	C12—C5—C6—C11	−179.24 (19)
C11—N4—C3—C2	−178.14 (16)	C4—C5—C6—C7	−177.62 (18)
C3—N4—C11—C6	−1.3 (3)	C12—C5—C6—C7	2.3 (3)
C3—N4—C11—C10	178.17 (17)	N4—C11—C10—C9	−178.52 (18)
N4—C3—C4—C5	0.0 (3)	C6—C11—C10—C9	0.9 (3)
C2—C3—C4—C5	179.24 (18)	C11—C6—C7—C8	0.1 (3)
C3—C4—C5—C6	−1.0 (3)	C5—C6—C7—C8	178.57 (19)
C3—C4—C5—C12	179.09 (19)	C11—C10—C9—C8	−0.8 (3)
N2—N3—C2—C3	−179.41 (16)	C11—C10—C9—C13	178.36 (19)
N4—C3—C2—N3	−174.01 (17)	C6—C7—C8—C9	0.1 (3)
C4—C3—C2—N3	6.7 (3)	C10—C9—C8—C7	0.2 (3)
N4—C11—C6—C7	178.87 (18)	C13—C9—C8—C7	−178.9 (2)
C10—C11—C6—C7	−0.6 (3)		

### Hydrogen-bond geometry (Å, º)

**Table:**

*D*—H···*A*	*D*—H	H···*A*	*D*···*A*	*D*—H···*A*
N1—H1*A*···O3^i^	0.86	2.08	2.9277 (18)	171
N2—H2···O1^ii^	0.86	2.01	2.867 (2)	175
O2—H2*B*···O3^i^	0.85	2.03	2.814	154
O2—H2*C*···O1	0.85	1.92	2.769	175
O3—H3*A*···O2	0.85	1.83	2.665	169
O3—H3*B*···N4^ii^	0.85	2.02	2.8706 (1)	176

Symmetry codes: (i) −*x*+5/2, *y*−1/2, −*z*+3/2; (ii) −*x*+2, −*y*+1, −*z*+1.
